# Circulating CTRP5 in rheumatoid arthritis: an exploratory biomarker study

**DOI:** 10.1186/s41927-022-00254-2

**Published:** 2022-04-25

**Authors:** Marjan Taherian, Simin Almasi, Saber Mehdizadeh, Banafshe Fazeli, Mahsa Kalantari, Paria Bayati, Salar Pashangzadeh, Nazanin Mojtabavi

**Affiliations:** 1 Department of Immunology, School of Medicine, Iran University of Medical Sciences, Tehran, Iran; 2 Immunology Research Center, Institute of Immunology and Infectious Diseases, Iran University of Medical Sciences, Tehran, Iran; 3 Rheumatology Research Center, Firuzgar Hospital, Iran University of Medical Sciences, Tehran, Iran

**Keywords:** Rheumatoid Arthritis, CTRP5, Adipokine, Inflammation, ILD

## Abstract

**Background:**

Rheumatoid arthritis (RA) is an inflammatory disease that is characterized by the overproduction of cytokines. Among the newly discovered cytokines are the adipokines which are primarily produced by and released from the adipose tissue and some immune cells, as well as synovial cells. they are involved in various immune responses and inflammatory processes. However, there are controversial data regarding the pro-inflammatory or anti-inflammatory effects of adipokines in different conditions. C1q/TNF-related protein 5 (CTRP5) is a newly identified adipokine and adiponectin paralogous protein, which has been shown to be correlated with inflammatory diseases. Accordingly, the present study was designed to investigate the serum levels of CTRP5 in RA patients and evaluate any possible alterations in comparison to healthy individuals.

**Methods:**

Serum CTRP-5 levels were measured in 46 patients and 22 healthy controls by ELISA. The demographic, laboratory, and clinical features of the patients were also evaluated in order to find any correlations.

**Results:**

Serum levels of CTRP-5 were significantly (*p* < 0.0001) higher in patients with RA (14.88 ± 25.55) compared to healthy controls (4.262 ± 2.374). There was a significant correlation between serum CTRP-5 levels and triglyceride (TG) (r: − 0.3010, p: 0.0498), as well as erythrocyte sedimentation rate (ESR) (r: 0.3139, p: 0.0457), C-reactive protein (CRP) (r: 0.5140, p: 0.0008), and the number of white blood cells (WBC) (r: 0.3380, p: 0.0307), which are considered as the markers indicating the extent of inflammation. Moreover, CTRP-5 was found to be correlated with interstitial lung disease (ILD) (r: 0.3416, p: 0.0385), a comorbidity associated with RA disease.

**Conclusion:**

This study demonstrated the increased level of circulating CTRP-5 in RA patients, which correlated with some inflammation-associated parameters and RA-associated comorbidities. Our observations suggest CTRP-5 as a putative inflammatory biomarker in RA, which may be useful besides the other disease-related markers.

## Background

Rheumatoid arthritis (RA) is the most common chronic inflammatory joint disease characterized by synovial hyperplasia and joint destruction [1]. The inflammation and joint destruction are mainly mediated by elevated levels of cytokines, chemokines, and matrix metalloproteinases [2–4]. The blockade of pro-inflammatory cytokines, such as interleukin (IL)-1, IL-6, and tumor necrosis factor (TNF)-α, has been a common medical approach for the treatment of RA [5]. Furthermore, increased production of the cytokine‐like mediators, called adipocytokines or adipokines, has recently attracted much interest [6]. However, the alterations of adipokines and their contribution to the pathogenesis of RA were not fully addressed.

Adipokines are some sort of protein mediators secreted by the adipose tissue, which is also among the major contributors of joint inflammation in RA. Although adipokines display both pro-inflammatory and anti-inflammatory roles, a growing body of evidence support an association between adipokines and inflammatory responses in several chronic inflammatory diseases [7, 8]. The first discovered adipokine, leptin, has been shown to be elevated in sera from RA patients with high disease activity [9]. In addition, upregulated resistin, another adipokine, in serum and synovial fluid from RA patients was observed to be correlated with disease activity [10]. Several studies have also demonstrated the increased levels of adiponectin, a well-known adipokine, both in circulation and synovial joint of RA patients; accordingly, a significant pro-inflammatory role was suggested for adiponectin in the pathophysiology of RA [11–13]. Adiponectin belongs to the C1q/TNF-related protein (CTRP) family, which also has 15 additional identified members (CTRP1-15) with shared structural homology. The members of this tightly conserved family are secreted plasma proteins that play diverse roles in metabolism and immunity [14, 15].

C1q/TNF-related protein 5 (CTRP5) is a 25 KDa secretory protein involved in the various metabolism functions as well as in immune responses. CTRP5 is expressed in many tissues, including adipose, myocyte, ciliary epithelium, liver, and retinal pigment; it has been reported that this adipokine is found in human serum [16–19]. A recent study demonstrated the positive correlation between the serum levels of CTRP5 and systemic inflammation in chronic obstructive pulmonary disease (COPD), suggesting that CTRP5 could have a role in inflammatory diseases [20]. so far, no study has been conducted to investigate the CTRP5 levels in sera from RA patients, as well as to find any possible correlations between CTRP5 and RA-associated parameters and comorbidities. Hence, this preliminary clinical study was designed to compare serum CTRP5 levels between RA and control groups and further investigate any possible correlation between CTRP5 and clinical/laboratory parameters of disease.

## Methods

### Study population

Fifty patients with active rheumatoid arthritis aged 27 to 81 years old from the Department of Rheumatology (Firouzgar Hospital, Tehran, Iran) were enrolled in this study. Also, 26 healthy individuals with no known history of any inflammatory or rheumatologic diseases, who were referred to the hospital laboratory for routine checkups and whose findings were normal were chosen as the control group. All participants filled out a written informed consent form confirmed by the ethics committee of the Iran University of medical sciences (ethics code: IR.IUMS.FMD.REC.1398.001). The body mass index (BMI) of subjects was calculated as weight/height^2^ (kg/m^2^). Disease activity score 28 (DAS28) was assessed as described previously [21]. In order to assess the joint erosion, hand x-rays of the patients were assessed by an expert rheumatologist who has detected and confirmed the presence of bone erosion in the hands. Moreover, the patients underwent computed tomography (CT) scan to evaluate ILD in the lungs. Radiographic findings identifying ILD, such as ground glass, honeycomb, and reticular, were reported by radiologists. Demographic features and laboratory findings of RA patients are provided in Table 1. Moreover, the gender distribution and comorbidities associated with RA patients are displayed in Table 2.

**Table 1:** Demographic and laboratory characteristics of RA patients

	Age	BMI	Disease duration	DAS28	CTRP5	RF	Anti-CCP	ESR	CRP	SGOT	SGPT	TG	HDL-C	LDL-C	WBC
Minimum	27.00	20.00	2.000	1.100	3.900	0.000	2.000	2.000	1.000	7.000	5.000	63.00	30.00	52.00	4000
Maximum	81.00	33.30	38.00	4.890	144.9	118.0	440.0	103.0	40.00	98.00	63.00	324.0	160.0	190.0	13,400
Range	54.00	13.30	36.00	3.790	141.0	118.0	438.0	101.0	39.00	91.00	58.00	261.0	130.0	138.0	9400
Mean	56.00	27.62	14.24	2.462	14.88	23.27	125.8	27.27	9.718	23.29	22.61	137.8	50.56	108.1	7500
Std. Deviation	12.22	3.207	9.620	0.8162	25.55	29.00	119.3	21.45	10.28	15.35	11.33	53.86	19.33	35.29	2105
Std. Error of Mean	1.782	0.5202	1.418	0.1324	3.851	5.048	22.55	3.349	1.646	2.397	1.770	8.028	3.095	5.652	317.4

**Table 2:** Gender distribution and comorbidities in RA group

Gender		ILD	Joint Erosion
Male	22.70%	35.90%	64.86%
Female	77.30%		

### Blood sampling and analysis

Venous blood samples were collected following overnight fasting. Blood samples were held at room temperature for at least 30 min to allow coagulation and then were centrifuged at 1500 × g for 15 min, then serum was separated immediately. Serum aliquots were stored frozen at − 80 °C until measurement of CTRP5.

### Biochemical and laboratory evaluation

Serum triglycerides (TG), high-density lipoprotein cholesterol (HDL-C), and total cholesterol were measured by an auto-analyzer. Low-density lipoprotein cholesterol (LDL-C) was calculated considering the following formula: LDL-C = total cholesterol—(HDL-C + triglycerides/5). The levels of alanine aminotransferase (ALT) and aspartate aminotransferase (AST) were also assessed by enzymatic colorimetric assays (Pars Azmoon kit, Tehran, Iran).

White blood cells (WBC) were evaluated using an automatic analyzer. Erythrocyte sedimentation rate (ESR) and C-reactive protein (CRP) were also evaluated.

### Measurement of circulating CTRP5 by ELISA

A commercially available human enzyme-linked immunosorbent assay (ELISA) kit (CTRP5 / C1QTNF5 ELISA kit, Aviscera Bioscience, USA) was utilized for the measurement of the circulating CTRP5 levels (in duplicate) according to the manufacturer's instructions.

### Statistical analysis

All the graphs and data analyses were performed with GraphPad Prism software version 8.0. Descriptive analysis was applied, and normality was tested using the Kolmogorov–Smirnov test for quantitative variables. A comparison of the different parameters between the two study groups was made by the independent t-test. For data not complying with normal distribution, the comparison between the two study groups was performed by the Mann–Whitney U test. The Receiver operating characteristic (ROC) curve was also plotted using GraphPad Prism software to indicate the sensitivity and specificity of CTRP5 in order to evaluate the diagnostic value of CTRP5 in RA. Associations between CTRP5 and other parameters were assessed using Pearson's and Spearman correlation coefficients for normal distributed and non-parametric data, respectively. Confidence intervals (CI) were calculated at 95%, and two-tailed p values < 0.05 were considered statistically significant.

## Result

### Demographic and laboratory characteristics of the study population

The laboratory findings and demographic features of the two study groups are provided in Table 3. The mean age and TG in RA patients were the same as in healthy controls, while significant differences were observed in BMI, CRP, ESR, and WBCs of the two groups.

**Table 3:** Comparison of the demographic and laboratory characteristics of control subjects and RA patients

Variables	Controls n = 22	RA patients n = 46	*p* values
Age, years	50.32 ± 11.98	55.93 ± 10.14	0.0636
BMI, kg/m^2^	25.35 ± 2.669	27.95 ± 2.998	0.0004*
CRP, mg/dl	1.074 ± 0.6841	9.718 ± 10.28	0.0057*
ESR, mm/h	5.667 ± 2.777	27.27 ± 21.45	< 0.0001*
TG, mg/dl	115.5 ± 40.91	116.0 ± 56.96	0.9733
WBC, × 10^9^/L	6.214 ± 0.7754	7.690 ± 2.025	0.0133*

Results are displayed as mean ± SD. The t-test and Mann–Whitney U test were used, and a p-value of < 0.05 was considered significant (*). BMI: body mass index; CRP: C-reactive protein; ESR: Erythrocyte sedimentation rate; TG: triglycerides; WBC: white blood cell

**Fig. 1:**
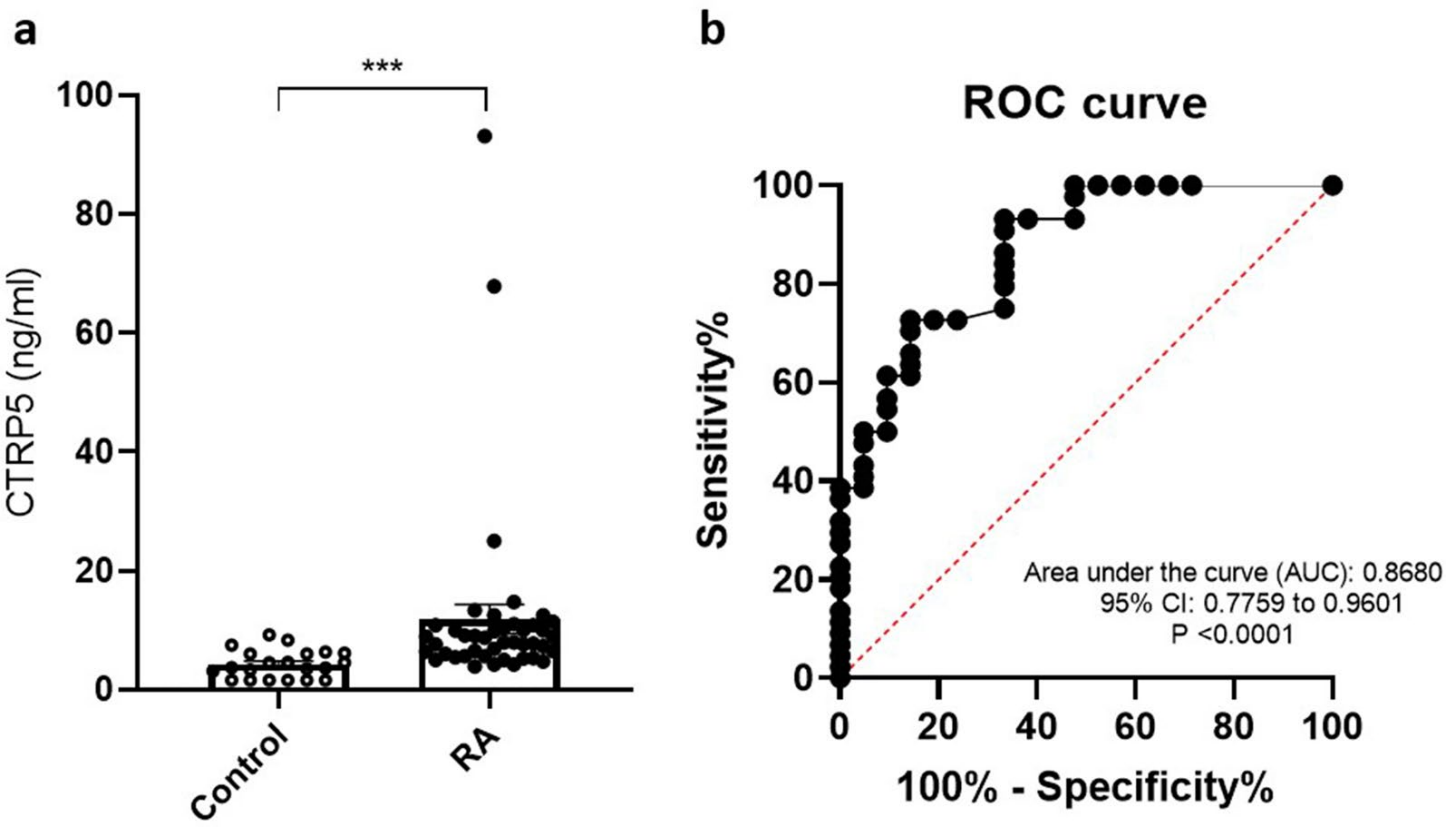
**a** Circulating levels of CTRP5 in both control and RA subjects. The data (without normal distribution) were analyzed by the Mann–Whitney U test and expressed as median ± SEM. **b** Receiver operating characteristic (ROC) curves for diagnosis RA patients by circulating CTRP5 levels. ***p < 0.0001, CTRP5: C1q/tumor necrosis-factor related protein-5

Comparing the serum levels of CTRP5 between the two studied groups demonstrated that the concentration of this adipokine is significantly higher in RA patients compared to the healthy controls (14.88 ± 25.55 in RA patients vs. 4.262 ± 2.374 in the controls (*p* < 0.0001) (Fig. 1a).

### Diagnostic value of serum CTRP5 levels in RA

ROC curve analysis of the serum levels of CTRP5 revealed that this adipokine is capable of efficiently differentiating the RA patients from the healthy controls with an acceptable AUC of 0.8680 as well as good sensitivity and specificity (Fig. 1b). The assigned cutoff value in this study was 6.350, which showed a sensitivity of 72.73%, and specificity of 85.71% (P-value < 0.0001).

### Correlation between CTRP5 and demographic, laboratory, and clinical data in RA patients

**Fig. 2:**
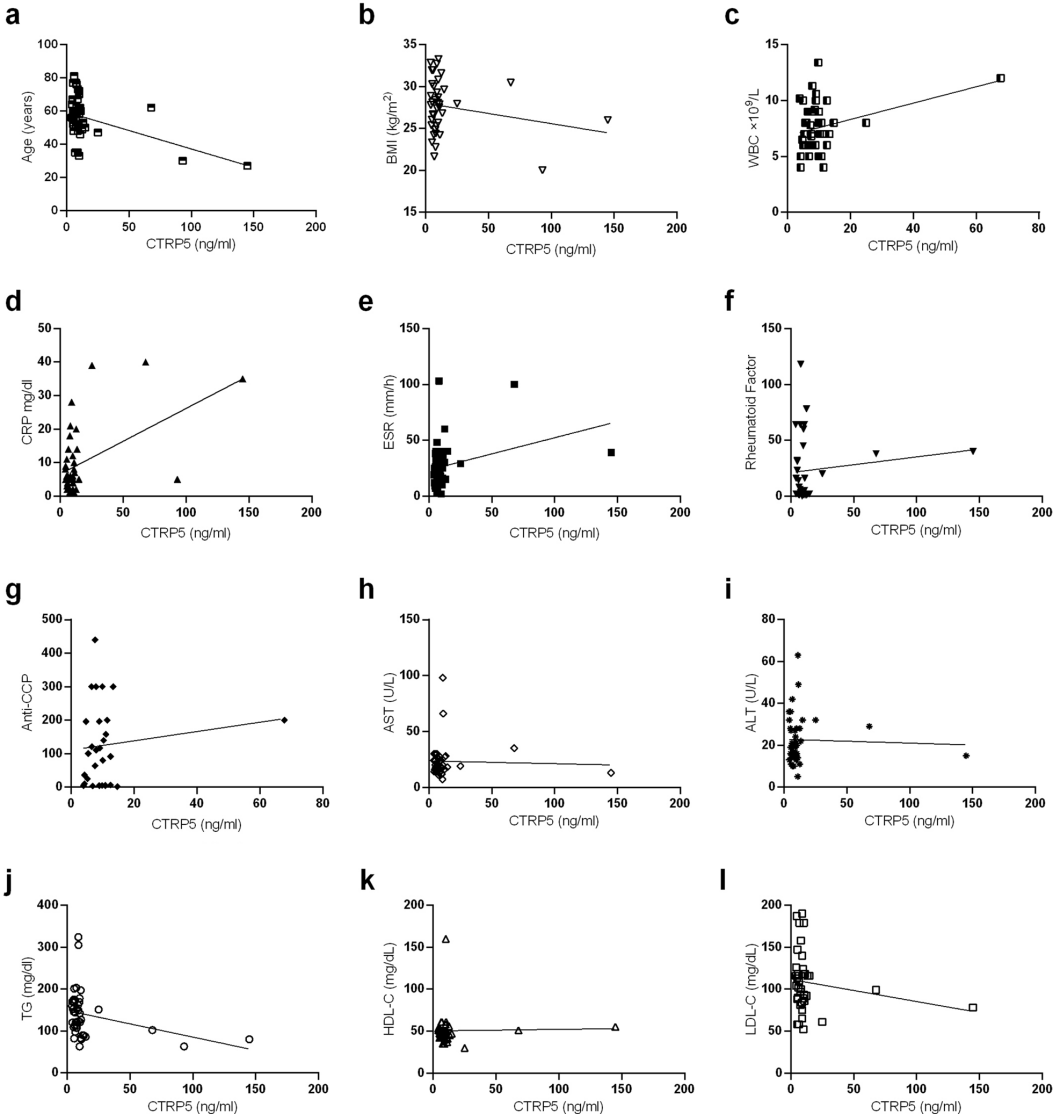
Graphic representation of correlations between circulating CTRP5 levels and Age (**a**), BMI (**b**), WBC (**c**), CRP (**d**), ESR (**e**), RF (**f**), Anti-CCP (**g**), AST (**h**), ALT (**i**), TG (**j**), HDL-C (**k**) and LDL-C (**l**) in RA patients. The corresponding correlation coefficient (r) and *p* values (p) are shown in Table 5. CTRP5: C1q/tumor necrosis-factor related protein-5; BMI: body mass index; WBC: white blood cell; CRP: C-reactive protein; ESR: Erythrocyte sedimentation rate; RF: Rheumatoid factor; Anti-CCP: Anti-cyclic citrullinated peptides; AST: aspartate aminotransferase; ALT: alanine aminotransferase; TG: triglycerides; HDL: high-density lipoprotein; LDL: low-density lipoprotein

**Fig. 3:**
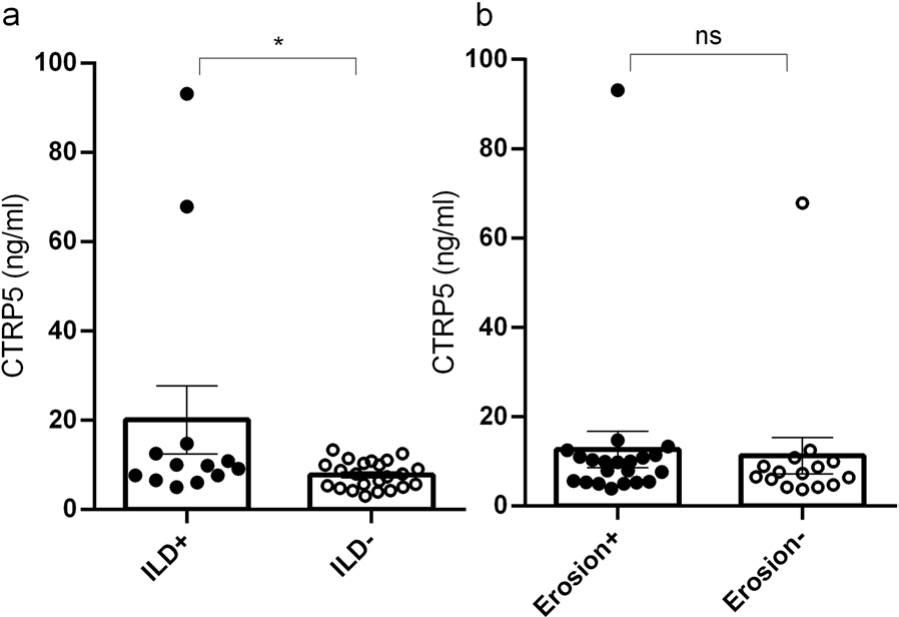
The circulating level of CTRP5 in RA patients with/without ILD (**a**) and erosion (**b**). The data expressed as median ± SEM, *p value < 0.05. CTRP5: C1q/tumor necrosis factor-related protein-5; ILD: Interstitial lung disease

Statistical analyses demonstrated that the rise of serum CTRP5 in RA patients is significantly correlated with a higher incidence of ILD (Table 4), as well as with increased WBC, CRP, and ESR (Table 5 and Fig. 2c–e). Also, a significant negative correlation was observed between serum CTRP5 of patients and their serum TG (Table 5 and Fig. 2j). Among the other clinical data of the patients that were considered in this study, increased serum CTRP5 was shown to be slightly correlated with decreased LDL-C, BMI; while no correlation was found between the serum CTRP5 and disease duration as well as joint erosion; nor it was correlated with the laboratory data such as RF, anti-CCP, ALT, AST, and HDL-C (Table 5 and Fig. 2). Based on the presence of ILD in RA patients, they were divided into two subgroups of ILD + and ILD-, and it was observed that serum CTRP5 was significantly higher in the ILD + group compared to ILD- group (Fig. 3a). Furthermore, the patients were divided based on the presence of joint erosion as well; it was found that serum CTRP5 was slightly higher in those who presented with joint erosion (Fig. 3b).

**Table 4:** Correlation coefficient between CTRP5 levels and clinical characteristics in the patients with RA

	CTRP5	
	r	*p*
Duration	− 0.1404	0.3751
DAS28	0.1331	0.4389
ILD	0.3416	0.0385*
Erosion	− 0.2269	0.1899

Pearson or Spearman correlation method was used for data with or without normal distribution, respectively. r: correlation coefficient; p: p values; *: a p-value of < 0.05 was considered significant. CTRP5: C1q/tumor necrosis factor-related protein-5; ILD: Interstitial lung disease

**Table 5:** The correlation coefficient between circulating CTRP5 and demographic or laboratory findings in the patients with RA

	CTRP5	
	r	*p*
Age	− 0.4561	− 0.4561
BMI	− 0.2078	0.2106
WBC	0.3380	0.0307*
CRP	0.5140	0.0008*
ESR	0.3139	0.0457*
RF	0.1245	0.4899
Anti-CCP	0.1342	0.4959
AST	− 0.03698	0.8184
ALT	− 0.03703	0.8182
TG	− 0.3010	0.0498*
HDL	0.02280	0.8904
LDL	− 0.1780	0.2782

Pearson or Spearman correlation method was used for data with or without normal distribution, respectively. r: correlation coefficient; p: *p* values; *: a *p* value of < 0.05 was considered significant. CTRP5: C1q/tumor necrosis factor-related protein-5; BMI: body mass index; WBC: white blood cell; CRP: C-reactive protein; ESR: Erythrocyte sedimentation rate; RF: Rheumatoid factor; Anti-CCP: Anti-cyclic citrullinated peptides; AST: aspartate aminotransferase; ALT: alanine aminotransferase; TG: triglycerides; HDL: high-density lipoprotein; LDL: low-density lipoprotein

## Discussion

In the current study, the serum levels of CTRP5 were measured in RA patients using ELISA and the results were further evaluated to find any possible correlations with other comorbidities and inflammatory markers associated with RA. We demonstrated for the first time that serum CTRP5 levels are altered in RA patients, which also was correlated with some related comorbidities and other inflammatory biomarkers as well. Furthermore, according to the ROC curve analysis, CTRP5 could be suggested as a good diagnostic biomarker in RA, which requires further evaluations in larger study populations with various ethnicities. The main findings of the current study are summarized as follows: (i) levels of circulating CTRP5 are significantly higher in RA patients in comparison to the healthy controls; (ii) the positive correlations were found between serum levels of CTRP5 and some parameters related to inflammation, including CRP, ESR and the number of WBC in patients with RA; (iii) inverse correlations were observed between serum levels of CTRP5 and some parameters of lipid metabolism, including TG and LDL-C in patients with RA; iv) an association was observed between CTRP5 levels and ILD involvement in RA patients.

To date, several serologic markers, such as CRP and ESR, as well as leukocyte elevation, have been commonly used to evaluate inflammation and disease activity in RA patients [22–24]. However, none of these inflammation-related markers were sufficient for definitive evaluations. Hence, several combinations of different markers have been suggested for more precision. The remarkable correlations observed between CTRP5 and the examined inflammatory markers here suggest that CTRP5 could be considered as a marker associated with inflammation in RA. Accordingly, CTRP5 can be helpful in combination with other parameters to improve disease evaluation in RA patients.

Moreover, ROC analysis demonstrated a reliable predictive value for serum concentrations of CTRP5 in the diagnosis of RA. However, CTRP5 levels showed no correlation with disease activity. It should be noted that disease activities among the majority of studied patients were well-controlled (The mean DAS28: 2.462 ± 0.8162, with maximum 4.89 and minimum 1.1) by receiving anti-rheumatic drugs (Table 6). Nevertheless, further studies are needed to assess the efficacy and ability of serum CTRP5 measurement for evaluating disease activity in RA. ILD is comorbidity found in RA patients, and it is associated with poor prognosis; besides, it is considered a strong predictor of mortality in RA patients [25]. We found that serum CTRP5 levels were related to the incidence of ILD in RA patients. Consistently, several studies have reported an association between adipokines and inflammatory lung diseases [26]. The circulating CTRP5 was shown to be associated with the extent of lung dysfunction and systemic inflammation in COPD patients [20]. The findings of the current study support an association between CTRP5 and interstitial lung diseases. However, the role of CTRP5 in the pathogenesis and prognosis of lung diseases remains to be elucidated. Our data revealed that CTRP5 levels were negatively correlated with TG significantly and with LDL-C weakly. This finding is consistent with some other reports concerning CTRP5 and lipid metabolism in the literature. For instance, an inverse correlation has been demonstrated between CTRP5 and TG in patients with type 2 diabetes and nonalcoholic fatty liver disease [27]. Furthermore, a weak negative correlation between CTRP5 and TG and a positive correlation with HDL-C was recently reported in women with polycystic ovary syndrome [28]. Whereas no correlation between circulating CTRP5 and HDL-C was detected, some shreds of evidence linked the upregulated CTRP5 to increased adipose tissue [29, 30]. Also, alterations in lipid profiles have been frequently reported in RA patients; However, the precise impact of adipokines on the lipid metabolism of these patients remains to be clarified [31–33].

**Table 6:** The drugs and the number of RA patients that received them during treatment

Treatment	Number of RA patients	Percent of RA patients (%)
Hydroxychloroquine	18	~ 40
Methotrexate	38	~ 83
Sulfasalazine	22	~ 48
Prednisolone	36	~ 79
AZARAM	1	~ 2
Biologic Treatment	1	~ 2
No Treatment	2	~ 4

The expression of CTRP5 in children's adipocytes was directly correlated to the degree of obesity and their age [29]. Conversely, several investigations identified an inverse correlation between CTRP5 levels and BMI [27, 28]. According to the present data, although BMI was significantly different between RA patients and healthy individuals, we observed a non-significant negative trend between circulating CTRP5 and BMI in RA patients. The underlying cause of these discrepancies is unknown, and elucidation of the relationship between serum CTRP5 concentration and anthropometric parameters requires further investigations with larger numbers of subjects to be examined.

The limitations of the present study are as follows: (a) the relatively small number of subjects; (b) the unbalanced distribution of disease severity and relatively mild RA in most of our patients (mean DAS28: 2.462, with maximum 4.890, and minimum 1.100); (c) The control individuals enrolled in the study, were chosen among healthy people (with normal laboratory findings) who were referred to the laboratory for routine checkups; as a result, further evaluations (retrospective) could not be performed after the study was finished; likewise, precise group matching between the controls and patients was not possible.

Addressing the above issues would be useful in future studies.

## Conclusion

It was found that the circulating levels of CTRP-5 are increased in RA patients, suggesting that this adipokine could be involved in the pathophysiology of RA. Our observations also demonstrated that CTRP-5 could be correlated with some RA-associated comorbidities as well as inflammatory biomarkers commonly utilized for assessing RA activity. These findings may represent the adipokine CTRP5 as a possible diagnostic biomarker for RA.

## Data Availability

The datasets generated and/or analysed during the current study are not publicly available due to limitations of ethical approval involving the patient data and anonymity but are available from the corresponding author on reasonable request.

## Abbreviations

RA: Rheumatoid arthritis
CTRP: C1q/TNF-related protein
IL: Interleukin
TNF: Tumor necrosis factor
COPD: Chronic obstructive pulmonary disease
BMI: Body mass index
DAS28: Disease activity score 28
HDL: High-density lipoproteins
LDL: Low-density lipoproteins
TG: Triglyceride
ALT: Alanine aminotransferase
AST: Aspartate aminotransferase
WBC: White blood cells
ESR: Erythrocyte sedimentation rate
CRP: C-reactive protein
ELISA: Enzyme-linked immunosorbent assay
ROC: Receiver operating characteristic
CI: Confidence interval
ILD: Interstitial lung disease
RF: Rheumatoid factor
